# Supplementary material for: Methane cycling microbes are important predictors of methylmercury accumulation in rice paddies
Source: Appl Environ Microbiol. 2026 Feb 17;92(3):e02028-25. doi: 10.1128/aem.02028-25 (PMC12997803; doi:10.1128/aem.02028-25)
Supplement: Supplemental material — Supplemental methods; Fig. S1 to S11. [file aem.02028-25-s0001.docx]

**Supplementary Materials**

**Methane cycling microbes are important predictors of methylmercury accumulation in rice paddies­**

Rui Zhang^a^, Alexandre J Poulain^a^, Qiang Pu^b^, Jiang Liu^b^, Mahmoud A. Abdelhafiz^b^, Xinbin Feng^b^, Bo Meng^b#^, Daniel S Grégoire^c#^

^a^ Department of Biology, University of Ottawa, Ottawa, Canada

^b^ State Key Laboratory of Environmental Geochemistry, Institute of Geochemistry, Chinese Academy of Sciences, Guiyang, China

^c^ Department of Chemistry, Carleton University, Ottawa, Canada

#Address correspondence to Daniel S Grégoire, [danielgregoire@cunet.carleton.ca](mailto:danielgregoire@cunet.carleton.ca) and Bo Meng: [mengbo@vip.skleg.cn](mailto:mengbo@vip.skleg.cn).

**Table of Contents**

[Supplementary Text 3](#_Toc205547259)

[Supplementary Method 1. 3](#_Toc205547260)

[Supplementary Method 2. 4](#_Toc205547261)

[Supplementary Method 3. 5](#_Toc205547262)

[Supplementary Method 4. 6](#_Toc205547263)

[Supplementary Discussion 1. 8](#_Toc205547264)

[Supplementary Discussion 2. 9](#_Toc205547265)

[Software Version Summary Table 11](#_Toc205547266)

[Reference 13](#_Toc205547267)

[Supplementary Figures 18](#_Toc205547268)

[Figure S1. 18](#_Toc205547269)

[Figure S2. 19](#_Toc205547270)

[Figure S3. 20](#_Toc205547271)

[Figure S4. 21](#_Toc205547272)

[Figure S5. 22](#_Toc205547273)

[Figure S6. 23](#_Toc205547274)

[Figure S7. 24](#_Toc205547275)

[Figure S8. 25](#_Toc205547276)

[Figure S9. 26](#_Toc205547277)

[Figure S10 27](#_Toc205547278)

[Figure S11. 28](#_Toc205547279)

# Supplementary Text

Supplementary Method 1. Soil collection, geochemical analyses, and sequencing

Sampling in rice fields was conducted in Guizhou province, Southwest China, from May 2021 to April 2022. The sampling locations included Huaxi (HX, 26°24'58.3"N 106°31'00.5"E), a regional background site; Gouxi (GX, 27°33'37.1"N 109°11'38.8"E), an artisanal Hg smelting area; and Sikeng (SK, 27°30'38.9"N 109°12'10.1"E), an abandoned Hg mining area. Specifically, samples collected on May 18, 2021, represent a pre-plantation time point before flooding. Rice planting began on June 9, 2021 (herein designated as day 0). Subsequent sampling was performed every 20 days during the rice growth period and every 30–60 days during the fallow period. To account for the varying sampling dates across sites, we provide an overview of the corresponding rice growth stages throughout the season as follows: days 0–20 correspond to the tillering stage, days 20–40 to the jointing stage, days 40–60 to the heading stage, and days 60–80 to the ripening stage. Notably, rice paddies remained flooded from pre-plantation until approximately day 60. By day 80 (September 1, 2021), fields were dried and ready for harvest.

Surface soil was defined as the layer 0-5 cm below the soil-water interface, specifically from areas without rice root attachment. Rhizosphere soil was obtained by carefully removing rice plants and collecting the soil directly adhering to the roots. Overlying water was sampled from the paddy surface using sterile syringes and filtered through 0.45 μm filters. Porewater was extracted from both surface (0-5 cm) and subsurface (15-20 cm) soil samples by centrifugation at 4000 rpm for 20 minutes. Surface and rhizosphere soils were collected from each site in triplicates. These replicates were combined into composite samples for downstream nucleic acid extraction. Samples designated for DNA extraction were stored at -20°C, while those intended for RNA extraction were stored at -80°C to preserve nucleic acid integrity. Additional soil samples were stored at -4°C for subsequent physiochemical parameter analyses.

Geochemical analyses were conducted at the Institute of Geochemistry, Chinese Academy of Sciences (CAS), experienced lab technicians. Soil samples were analyzed for multiple chemical parameters using established extraction and measurement protocols. Anions (NO₃⁻, SO₄²⁻, Cl⁻, and F⁻) were extracted by mixing soil with ultrapure deionized water in a 1:10 ratio (w/v), followed by one hour of shaking, 10 minutes of centrifugation, and filtration through 0.22 μm pore-size filters before analysis via ion chromatography (ICS90, DIONEXm, USA). Elemental sulfur (S⁰) in soil was measured using high-performance liquid chromatography (HPLC) equipped with a UV-Vis absorption detector (Shimadzu SPD-20A, Japan) . Dissolved organic matter (DOM), represented as water-soluble dissolved organic carbon (DOC), was extracted in Milli-Q water at a 1:10 ratio (w/v), filtered with 0.45 μm filters, and quantified using a total organic carbon analyzer (Vario TOC cube, Elementar) (1). Soil organic matter (SOM) content was estimated using the loss on ignition (LOI) method (2). Total mercury (THg) content was quantified by digesting approximately 0.2 g of soil in 5 mL of freshly prepared aqua regia (HCl: HNO₃ = 3:1, v/v) and 5 mL of Milli-Q water at 95 °C for 55 minutes; THg in the digest solution was then measured with cold vapor atomic fluorescence spectrometry (CVAFS, Brooks Rand Model III, USA) per USEPA method 1631. Methylmercury (MeHg) analysis involved extracting 0.3-0.4 g of soil with a CuSO₄-methanol solution, followed by gas chromatography-CVAFS (GC-CVAFS, Brooks Rand Model III, USA) per USEPA method 1630. To measure S²⁻, Fe²⁺ and Fe^3+^ concentrations in porewater and overlaying water, the collected water samples were first centrifuged at 3500 × g for 15 min. S²⁻ was determined using the methylene blue method, with a detection limit of 0.13 μM (3). Fe²⁺ and Fe^3+^ were determined via the ferrozine method with a detection limit of 10 μM (4). Overlaying water ORP were measured in-situ using a YSI ProQuatro Multiparameter Meter (Xylem Analytics, USA). Detailed geochemical property measurements are provided in Table S8.

DNA extractions were carried out using the PowerSoil DNA Isolation Kit following the manufacturer’s instructions. Metagenomic sequencing were performed on an Illumina NovaSeq 6000 platform, producing 150 bp paired-end reads. Sequencing was conducted at Azenta (Suzhou, China). The sequencing aimed for a read depth of approximately 15 Gbp per sample (range: 10.9 – 20 Gbp, Table S9). In total, 50 metagenomic samples were obtained.

Supplementary Method 2. Genome assembly and binning

Trimming and adaptor removal of paired-end DNA reads were conducted using fastp (ver. 0.23.1) (5) with default parameters to retain reads exceeding a mean quality score threshold of Q20. Quality control statistics for these filtered reads were assessed using FastQC (ver. 0.11.9) (6) (Table S10). Individual assemblies of DNA reads from each sample were performed with MetaSPAdes (ver. 3.15.5) (7), employing default k-mer parameters (Table S11). This method was chosen for its ability to produce longer contigs, facilitating more complete gene recovery at the contig level. For co-assembly of reads from identical sites and soil types (HX_surface, HX_rhizosphere, GX_surface, GX_rhizosphere, SK_surface, SK_rhizosphere), we used MEGAHIT (ver. 1.2.9) (8) with custom parameters (--no-mercy, --presets meta-large, --kmin-1pass), selected for its computational efficiency in processing large datasets. Contigs from each co-assembly were initially trimmed to retain those exceeding 2000 bp using the 'anvi-script-reformat-fasta' command in Anvi’o (ver. 7.1) (9). Subsequently, metagenomic short reads from each sample were aligned to the co-assemblies using BWA-MEM (ver. 0.7.17) (10) and SAMtools (ver. 1.17) (11) to generate BAM files. The 'anvi-init-bam' function was then employed for sorting and indexing these BAM files, preparing them for binning algorithms. Co-assemblies were processed through four binning tools: MetaBAT 2 (ver. 2.15) (12), MaxBin 2 (ver. 2.2.7) (13), CONCOCT (ver. 1.1.0) (14), and VAMB (ver. 4.1.1) (15), utilizing BAM files from corresponding samples. This step yielded 5307 initial bins. Bins were evaluated for completeness and contamination using CheckM2 (ver. 1.0.2) (16). Given CheckM2's novelty compared to its predecessor, CheckM (17), a comparative analysis was conducted. Results indicated a significant positive correlation between the two in terms of quality metrics, with CheckM2 exhibiting a tenfold increase in processing speed compared to CheckM. The highest quality bins from each co-assembly were selected using DAS-Tool (ver. 1.1.6) (18). Subsequently, dRep (v3.0.0) (19) was applied to further dereplicate these bins with an ANI threshold of 98%. We used the parameters *--multiround_primary_clustering* and *--run_tertiary_clustering* to prevent scaffold overlap across multiple bins. We then manually refined the bins using 'anvi-refine' in Anvi’o, leveraging tetranucleotide frequency, differential coverage signals, and taxonomic information. This manual refinement resulted in 267 metagenome-assembled genomes (MAGs) with over 50% completeness and less than 10% contamination. Taxonomy was assigned to MAGs using GTDB-Tk (ver. 2.3.0) (20) based on GTDB release 214.

The binning pipeline prioritized co-assembly to leverage differential coverage signals from the time-series metagenomic dataset, enhancing the identification of low-abundance populations. However, this approach may encounter limitations in resolving strain-level variability (21). To address these potential limitations, an individual-assembly approach, wherein bins were formed using assemblies from individual metagenomes, was also implemented. This method yielded 201 metagenome-assembled genomes (MAGs) with completeness over 50% and contamination below 10%. Subsequent taxonomic classification of these 201 MAGs using GTDB revealed diversity at the class and family levels, encompassing 21 unique classes and 40 unique families (Table S1). In contrast, the co-assembly approach resulted in a broader diversity, identifying 57 unique classes and 117 unique families (Table S12). Based on these outcomes, the co-assembly approach was selected for further analysis due to its higher yield and greater diversity of MAGs. MAGs were assigned identifiers with prefixes indicating the site, binning algorithm origin and a number (e.g., HX-MT-MAG-01).

Supplementary Method 3. Methane-cycling, Hg-methylation and demethylation microbial abundance analyses

We identified Open reading frames (ORFs) on these contigs using Prodigal (ver. 2.6.3) (22), with outputs in GFF format. We mapped the metagenomic short reads to contigs using BWA MEM (ver. 0.7.17) (10). We used the featureCounts tool from the Subread package (ver. 2.0.4) (23) in paired-end mode with the --countReadPairs parameter to quantify reads mapped to each ORF. Functional genes representative of the guilds within contigs were retrieved using Hidden Markov Models (HMMs) from various databases through hmmsearch of the HMMER software (ver. 3.3.1) (24). Specifically, the McrA HMM (PF02249) was sourced from PFAM, while PmoA and MmoX HMMs were obtained from the GraftM gene packages (<https://data.ace.uq.edu.au/public/graftm/7>) with an E-value threshold of 1×10⁻⁵.

We conducted protein homology searches using BLASTp (ver. 2.14) against the NCBI non-redundant database (as of Dec 5, 2023) to confirm their identity and refine search results. Queries consisted of HMM hits of the corresponding genes, each limited to the single best hit (-max_target_seqs 1) to focus on the most relevant homolog. McrA, PmoA, and MmoX hits were filtered based on sequence titles indicative of their respective functions (e.g., McrA hits containing "methyl coenzyme M reductase"; PmoA and MmoX hits classified as "methane monooxygenase"). Extra precautions were taken with methane-cycling genes, as the PmoA sequence is homologous to other alkane monooxygenases, and PmoA is homologous to the AmoA of ammonia oxidizers. The highest similarity to a known methanotrophic isolate was prioritized.

HgcAB were identified using HMMs from the Hg-MATE-Db (ver. 1.01142021) (25) with E-values of 1×10⁻⁵⁰ and 1×10⁻³⁰, respectively. To eliminate paralogs, aligned HgcA and HgcB sequences were examined for the presence of the conserved putative cap helix motif [N(V/I)WCA(A/G)GK] and the conserved cysteine motif [C(M/I)ECGA], respectively, as previously reported (26). Similarly, MerB sequences were retrieved using a pre-compiled HMM (27), with sequence alignments inspected for characteristic sequence signatures: cysteine at position 96 (referenced to MerB from *R831b*), aspartic acid at position 99, and cysteines at positions 159 and 117 (referenced to MerB from the *Escherichia coli* plasmid [AAB49639.1]) (28).

Taxonomic classification of protein-coding genes was conducted using the MMseqs2 (ver. 2.15) taxonomy module (29). A database (seqTaxDB) was created using GTDB (release 214) (30), against which filtered HMM hits (queryDB) were searched for taxonomic assignment, employing a lowest common ancestor strategy of all equal scoring top hits (mmseqs taxonomy --lca-mode 4) at phylum and family ranks. Post-classification, sequences corresponding to known methanogens and anaerobic methanotrophs (for McrA) and aerobic/intro-aerobic methanotrophs (for PmoA and MmoX) were retained. Coverage values assigned to sequences from featureCounts were aggregated based on taxonomic classification to create gene-based community profiles.

Supplementary Method 4. Statistical analyses

Principal Component Analysis (PCA) of geochemical variables was performed using the prcomp function in the stats package (v4.3.1), with data standardized by scaling. Variance explained by each principal component and variable contributions were analyzed through PCA loadings and scores, with results presented in biplots color-coded by site.

Distance-based redundancy analysis (db-RDA) of methane-cycling communities was conducted using the capscale function in the vegan package (v2.6-6.1) (31) based on Bray-Curtis dissimilarity indices. Environmental variables including total mercury (THg), methylmercury (MeHg), pH, dissolved organic matter (DOM), DOM aromaticity (DOM SUVA_254_), nitrate, and sulfate were evaluated as potential constraining factors. Prior to ordination analysis, multicollinearity among environmental variables was assessed using variance inflation factors (VIF) through the vif function in the car package (v3.1-3) (32) applied to a linear model with THg as the response variable. All environmental variables exhibited VIF values below 2.2 (range: 1.03-2.16), indicating minimal multicollinearity and supporting the inclusion of all variables in the constrained ordination model. Overall significance of constrained variables and their individual contributions were assessed using anova.cca (31). Permutational multivariate ANOVA (PERMANOVA) was performed using the adonis function (31) with Bray-Curtis dissimilarity to assess effects of site and soil type on community abundance, with pairwise comparisons conducted using pairwise.adonis (<https://github.com/pmartinezarbizu/pairwiseAdonis>) and Bonferroni correction. Statistical differences in individual methane-cycling guild abundances and geochemical parameters across sites were evaluated using Kruskal-Wallis tests followed by pairwise Wilcoxon rank-sum tests.

To evaluate the relative importance of methylation potential (*hgcA* abundance) and mercury bioavailability (F1-Hg) in predicting MeHg concentrations, we employed multiple linear regression with formal model selection using Akaike's Information Criterion corrected for small sample sizes (AICc) via the AICcmodavg package. Seven candidate models representing different hypotheses were fitted, including single-predictor models, additive effects, interactive effects, and models controlling for site and compartment variation (Supplementary Result 2). Model assumptions were verified through statistical tests: normality of residuals (Shapiro-Wilk), homoscedasticity (Breusch-Pagan), independence of errors (Durbin-Watson), and multicollinearity (generalized variance inflation factors). All assumptions were satisfied for selected models. Similar multiple regression approaches were applied to assess relative importance of other functional genes.

MAG relative abundances were calculated by aligning reads to dereplicated genome sets using CoverM (v0.7.0) (coverm genome -m relative_abundance). Time-dependent correlations between MAG abundance and MeHg concentrations were identified using extended Local Similarity Analysis (eLSA v1.0) (33,34) in Python 2.7 with NumPy and SciPy (v1.2.3). eLSA was performed separately for each site (HX, GX, SK surface soils) across 11 time points with the following parameters: synchronous correlations only (maximum delay = 0), percentile normalization followed by Z-normalization, single replicate, and disabled bootstrap. MeHg data were log-transformed prior to analysis. Statistical significance was determined using Local Similarity scores and Spearman's rank correlations. False Discovery Rate (FDR) correction was applied using the Benjamini-Hochberg procedure, performed separately for each MAG rather than globally. For MAGs with fewer than 4 associations, FDR correction was omitted to prevent over-correction of limited data. Significant associations were defined as FDR-adjusted p < 0.05 for both eLSA and Spearman correlations, excluding insufficient data (LS = 1) and perfect correlations (LS = ±1). Networks were visualized using Gephi (v0.10.1) with Yifan Hu layout, and topological metrics were calculated using built-in statistics tools.

Random forest regression models were developed using R (randomForest, caret, rfPermute packages) to predict MeHg concentrations from 12 predictor variables: 9 microbial functional gene abundances (total *hgcA*, guild-specific *hgcA* variants, *mcrA*, *merB*, *pmoA*/*mmoX*, SRB, IRB) and 3 environmental parameters (F1-Hg, DOM, DOM SUVA_254_). We implemented 100-fold cross-validation using random 70:30 train-test splits across all data from the three sampling sites and multiple time points. Each Random Forest model used 2001 trees, mtry=4, and 100 permutation tests for variable importance significance. Model performance was evaluated using RMSE, MAE, R², and Pearson correlation coefficients, with confidence intervals calculated across all splits.

All analyses were conducted in R (v4.3.0) unless otherwise specified. Statistical significance was set at α = 0.05 for all tests.

Supplementary Discussion 1. Implications of ANME to Hg Cycling in rice paddies

We recovered sequences from *Methanoperedenaceae*, a group of anaerobic methanotrophs (ANME), during our *mcrA* analyses. These microorganisms perform anaerobic oxidation of methane (AOM) via reverse methanogenesis and displayed significantly higher abundance at mining-impacted sites, particularly SK (Fig. S9; Supplementary Result 5). This finding contrasts sharply with previous studies in Korean rice paddies, where ANME were rarely detected (35). At the transcriptomic level, we detected limited expression of both *mcrA* and *hgcA* genes associated with *Methanoperedenaceae* at mining-impacted sites (Fig. S4), suggesting potential metabolic activity, though this does not confirm Hg methylation capacity.

ANME can oxidize methane using various electron acceptors, including sulfate (in conjunction with syntrophic partners), nitrate (NO₃⁻), iron (Fe³⁺), and manganese (Mn⁴⁺), some of which were present at physiologically relevant concentrations at our sites (Fig. S1) (36–39). This suggests that ANME may contribute to nutrient cycling under the reduced conditions characteristic of Hg-contaminated paddies. Supporting this notion, we detected *Methanoperedenaceae*-associated *hgcA* sequences (Supplementary Result 7), which were sporadically recovered in GX and SK rhizosphere samples during days 40 to 80 (Fig. 2A) and resembled those found in previously reported ANME metagenome-assembled genomes (MAGs) (40,41). However, while these *hgcA*-like sequences hint at a potential link to Hg methylation, it is crucial to clarify that the ability of ANME to methylate Hg has not been experimentally confirmed.

Recent evidence suggests that the methyl group for MeHg production in methanogens originates from the Wolfe cycle rather than the acetyl-CoA pathway (42). Since ANME perform reverse methanogenesis involving methyl-group transfer, we hypothesize they may utilize this pathway for Hg methylation, a proposition requiring empirical validation, such as transcriptomic evidence, isotopic tracing studies, or cultivation-based experiments, to substantiate the potential role of ANME in Hg cycling.

From a theoretical thermodynamic perspective, *Methanoperedenaceae* could potentially couple AOM with Hg(II) reduction (Hg^2+^/Hg^0^, E^∘^≈0.80 V). The mercuric reductase gene (*merA*) is found in the genome of ANME taxa *Methanoperedens nitroreducens* (NCBI accession: WP_048089931.1) and AOM coupled to Hg(II) reduction could yield more energy than using iron (Fe^3+^/Fe^2+^, E^∘^≈0.77 V) or nitrate (NO^3-^/NO^2-^, E^∘^≈0.43 V) as electron acceptors, though less than with manganese (MnO₄⁻/Mn²⁺, E^∘^ ≈1.51 V), under standard conditions. The multi-heme cytochrome "wires" these archaea possess enable electron transfer to solid substrates (43), raising a possibility: could these microbes directly reduce Hg in Hg(II)-containing minerals present in rice paddy environments? This potential for Hg reduction and methylation, coupled with their distribution alongside methanogens, suggests ANME may play a dual role, serving as potential methane sinks in anoxic paddy environments while significantly impacting Hg cycling and bioavailability in these agricultural ecosystems. However, we must exercise caution regarding the assertion that ANME act as novel Hg methylators. Functional validation through controlled laboratory experiments, transcriptomic analyses, isotopic tracing studies, and cultivation-based approaches are essential prerequisites before drawing any mechanistic conclusions about ANME involvement in environmental Hg cycling.

Supplementary Discussion 2. Network property explanation

We employed genome-resolved metagenomics and co-occurrence networks (Fig. S10, S11) to elucidate specific microbial populations associated with MeHg cycling. By reconstructing metagenome-assembled genomes (MAGs) and linking their abundance with MeHg concentration through networks, we established associations between distinct microbial populations with observed MeHg dynamics in situ (Table S6, S7).

The microbial networks at HX, GX, and SK exhibit distinct structural characteristics, as evidenced by differences in network density, modularity, and average node connectivity (Fig. S11). The HX and GX networks display relatively high densities of 38.9% and 34.8%, respectively, indicating a more interconnected community structure, with nodes (microbial genomes) having a higher average number of neighbors (32.6 for HX and 25.4 for GX). These networks also have lower modularity scores (0.148 and 0.115), suggesting a less compartmentalized and more homogeneous community where microbial species are more interconnected. In contrast, the SK network shows a more modular structure (modularity: 0.38), indicating that the community is divided into more distinct subgroups with specialized interactions. However, SK has the lowest network density at 20.3% and fewer neighbors per node (21.2), suggesting less overall connectivity and possibly a more specialized or niche-driven network structure. Despite these structural differences, the percentage of positive edges (PEP) across all three networks is comparable (53.6%, 51.6%, and 59.11%), indicating that the balance of positive (e.g., cross-feeding, biofilm formation, or niche overlap) versus negative (e.g., prey-predation relationships, competition) interactions is consistent across these environments (44). This consistency in PEP suggests that while the networks differ in overall structure and connectivity, the nature of microbial interactions within each network remains stable (45).

## Software Version Summary Table

| **Analysis Section** | **Software/Tool** | **Version** | **Purpose** |
| --- | --- | --- | --- |
| **Quality Control & Assembly** |  |  |  |
|  | fastp | 0.23.1 | Read trimming and quality filtering |
|  | FastQC | 0.11.9 | Quality control assessment |
|  | MetaSPAdes | 3.15.5 | Individual metagenomic assembly |
|  | MEGAHIT | 1.2.9 | Co-assembly of metagenomic reads |
|  | BWA-MEM | 0.7.17 | Read alignment to contigs |
|  | SAMtools | 1.17 | BAM file processing |
| **Genome Binning & Analysis** |  |  |  |
|  | Anvi'o | 7.1 | Bin Refinement |
|  | MetaBAT 2 | 2.15 | Metagenomic binning |
|  | MaxBin 2 | 2.2.7 | Metagenomic binning |
|  | CONCOCT | 1.1.0 | Metagenomic binning |
|  | VAMB | 4.1.1 | Metagenomic binning |
|  | CheckM2 | 1.0.2 | Bin quality assessment |
|  | DAS-Tool | 1.1.6 | Bin selection and refinement |
|  | dRep | 3.0.0 | Bin dereplication |
|  | GTDB-Tk | 2.3.0 | Taxonomic classification |
|  | GTDB | Release 214 | Taxonomic classification |
| **Functional Gene Analysis** |  |  |  |
|  | Prodigal | 2.6.3 | Open reading frame prediction |
|  | Subread (featureCounts) | 2.0.4 | Read quantification |
|  | HMMER | 3.3.1 | HMM-based gene identification |
|  | BLASTp | 2.14 | Protein homology searches |
|  | MMseqs2 | 2.15 | Taxonomic classification |
|  | Hg-MATE-Db | 1.01142021 | Mercury-cycling gene identification |
| **RNA Processing** |  |  |  |
|  | FLASH | 1.2.11 | Paired-end read merging |
|  | SortMeRNA | 4.3.6 | rRNA/non-rRNA classification |
| **Statistical Analysis** |  |  |  |
|  | R | 4.3.0 | Statistical analyses platform |
|  | vegan | 2.6-6.1 | Ecological statistical analysis |
|  | stats | 4.3.1 | Principal components analysis |
|  | car | 3.1-3 | Variance inflation factors |
|  | edgeR | 3.42.4 | Normalization and differential analysis |
|  | randomForest | 4.7-1.1 | Machine learning predictions |
|  | caret | 7.0.1 | Classification and regression training |
|  | rfPermute | 2.5.2 | Random forest permutation tests |
|  | AICcmodavg | 2.3-4 | Model selection |
| **Network & Correlation Analysis** |  |  |  |
|  | CoverM | 0.7.0 | MAG abundance calculation |
|  | eLSA | 1.0 | Extended Local Similarity Analysis |
|  | Gephi | 0.10.1 | Network visualization |

## Reference

1. Abdelhafiz MA, Liu J, Jiang T, Pu Q, Aslam MW, Zhang K, et al. DOM influences Hg methylation in paddy soils across a Hg contamination gradient. Environmental Pollution. 2023 Apr 1;322:121237.

2. Nayak AK, Rahman MM, Naidu R, Dhal B, Swain CK, Nayak AD, et al. Current and emerging methodologies for estimating carbon sequestration in agricultural soils: A review. Science of the total environment. 2019;665:890–912.

3. Cline JD. Spectrophotometric determination of hydrogen sulfide in natural waters 1. Limnology and Oceanography. 1969;14(3):454–8.

4. Viollier E, Inglett PW, Hunter K, Van Cappellen P. The ferrozine method revisited: Fe(II)/Fe(III) determination in natural waters. Applied Geochemistry. 2000 July 1;15(6):785–90.

5. Chen S, Zhou Y, Chen Y, Gu J. fastp: an ultra-fast all-in-one FASTQ preprocessor. Bioinformatics. 2018 Sept 1;34(17):i884–90.

6. Andrews S. Babraham Bioinformatics - FastQC A Quality Control tool for High Throughput Sequence Data [Internet]. 2010 [cited 2021 June 8]. Available from: http://www.bioinformatics.babraham.ac.uk/projects/fastqc/

7. Nurk S, Meleshko D, Korobeynikov A, Pevzner PA. metaSPAdes: a new versatile metagenomic assembler. Genome Res. 2017 May;27(5):824–34.

8. Li D, Liu CM, Luo R, Sadakane K, Lam TW. MEGAHIT: an ultra-fast single-node solution for large and complex metagenomics assembly via succinct de Bruijn graph. Bioinformatics. 2015 May 15;31(10):1674–6.

9. Eren AM, Kiefl E, Shaiber A, Veseli I, Miller SE, Schechter MS, et al. Community-led, integrated, reproducible multi-omics with anvi’o. Nat Microbiol. 2021 Jan;6(1):3–6.

10. Li H. Aligning sequence reads, clone sequences and assembly contigs with BWA-MEM. 2013 Mar 16 [cited 2022 Feb 4]; Available from: http://arxiv.org/abs/1303.3997v2

11. Li H, Handsaker B, Wysoker A, Fennell T, Ruan J, Homer N, et al. The Sequence Alignment/Map format and SAMtools. Bioinformatics. 2009 Aug 15;25(16):2078–9.

12. Kang DD, Li F, Kirton E, Thomas A, Egan R, An H, et al. MetaBAT 2: an adaptive binning algorithm for robust and efficient genome reconstruction from metagenome assemblies. PeerJ [Internet]. 2019 July 26 [cited 2020 Nov 19];7. Available from: https://www.ncbi.nlm.nih.gov/pmc/articles/PMC6662567/

13. Wu YW, Simmons BA, Singer SW. MaxBin 2.0: an automated binning algorithm to recover genomes from multiple metagenomic datasets. Bioinformatics. 2016 Feb 15;32(4):605–7.

14. Alneberg J, Bjarnason BS, de Bruijn I, Schirmer M, Quick J, Ijaz UZ, et al. Binning metagenomic contigs by coverage and composition. Nat Methods. 2014 Nov;11(11):1144–6.

15. Nissen JN, Johansen J, Allesøe RL, Sønderby CK, Armenteros JJA, Grønbech CH, et al. Improved metagenome binning and assembly using deep variational autoencoders. Nat Biotechnol. 2021 May;39(5):555–60.

16. Chklovski A, Parks DH, Woodcroft BJ, Tyson GW. CheckM2: a rapid, scalable and accurate tool for assessing microbial genome quality using machine learning. Nat Methods. 2023 Aug;20(8):1203–12.

17. Parks DH, Imelfort M, Skennerton CT, Hugenholtz P, Tyson GW. CheckM: assessing the quality of microbial genomes recovered from isolates, single cells, and metagenomes. Genome Res. 2015 July;25(7):1043–55.

18. Sieber CMK, Probst AJ, Sharrar A, Thomas BC, Hess M, Tringe SG, et al. Recovery of genomes from metagenomes via a dereplication, aggregation and scoring strategy. Nat Microbiol. 2018 July;3(7):836–43.

19. Olm MR, Brown CT, Brooks B, Banfield JF. dRep: a tool for fast and accurate genomic comparisons that enables improved genome recovery from metagenomes through de-replication. ISME J. 2017 Dec;11(12):2864–8.

20. Chaumeil PA, Mussig AJ, Hugenholtz P, Parks DH. GTDB-Tk: a toolkit to classify genomes with the Genome Taxonomy Database. Hancock J, editor. Bioinformatics. 2019 Nov 15;btz848.

21. Vosloo S, Huo L, Anderson CL, Dai Z, Sevillano M, Pinto A. Evaluating de Novo Assembly and Binning Strategies for Time Series Drinking Water Metagenomes. Microbiology Spectrum. 2021 Nov 3;9(3):e01434-21.

22. Hyatt D, Chen GL, LoCascio PF, Land ML, Larimer FW, Hauser LJ. Prodigal: prokaryotic gene recognition and translation initiation site identification. BMC Bioinformatics. 2010 Mar 8;11(1):119.

23. Liao Y, Smyth GK, Shi W. featureCounts: an efficient general purpose program for assigning sequence reads to genomic features. Bioinformatics. 2014 Apr 1;30(7):923–30.

24. Eddy SR. HMMER User’s Guide Biological sequence analysis using profile hidden Markov models. 2020 Nov;227.

25. Gionfriddo C, Capo E, Peterson B, Heyu Lin, Jones D, Bravo AG, et al. Hg-MATE-Db.v1.01142021 [Internet]. The Smithsonian Institution; 2021 [cited 2021 Jan 31]. p. 8700889 Bytes. Available from: https://smithsonian.figshare.com/articles/dataset/Hg-MATE-Db_v1_01142021/13105370

26. Parks JM, Johs A, Podar M, Bridou R, Hurt Jr RA, Smith SD, et al. The genetic basis for bacterial mercury methylation. Science. 2013;339(6125):1332–5.

27. Christakis CA, Barkay T, Boyd ES. Expanded Diversity and Phylogeny of mer Genes Broadens Mercury Resistance Paradigms and Reveals an Origin for MerA Among Thermophilic Archaea. Frontiers in Microbiology [Internet]. 2021 [cited 2022 Jan 13];12. Available from: https://www.frontiersin.org/article/10.3389/fmicb.2021.682605

28. Lafrance-Vanasse J, Lefebvre M, Di Lello P, Sygusch J, Omichinski JG. Crystal Structures of the Organomercurial Lyase MerB in Its Free and Mercury-bound Forms: INSIGHTS INTO THE MECHANISM OF METHYLMERCURY DEGRADATION*. Journal of Biological Chemistry. 2009 Jan 9;284(2):938–44.

29. Steinegger M, Söding J. MMseqs2 enables sensitive protein sequence searching for the analysis of massive data sets. Nat Biotechnol. 2017 Nov;35(11):1026–8.

30. Parks DH, Chuvochina M, Chaumeil PA, Rinke C, Mussig AJ, Hugenholtz P. A complete domain-to-species taxonomy for Bacteria and Archaea. Nat Biotechnol. 2020 Sept;38(9):1079–86.

31. Oksanen J, Blanchet FG, Friendly M, Kindt R, Legendre P, McGlinn D, et al. vegan: Community Ecology Package [Internet]. 2020. Available from: https://CRAN.R-project.org/package=vegan

32. Fox J, Weisberg S, Price B. car: Companion to Applied Regression [Internet]. 2001 [cited 2025 Sept 26]. p. 3.1-3. Available from: https://CRAN.R-project.org/package=car

33. Ruan Q, Dutta D, Schwalbach MS, Steele JA, Fuhrman JA, Sun F. Local similarity analysis reveals unique associations among marine bacterioplankton species and environmental factors. Bioinformatics. 2006 Oct 15;22(20):2532–8.

34. Xia LC, Steele JA, Cram JA, Cardon ZG, Simmons SL, Vallino JJ, et al. Extended local similarity analysis (eLSA) of microbial community and other time series data with replicates. BMC Syst Biol. 2011 Dec 14;5(2):S15.

35. Lee HJ, Kim SY, Kim PJ, Madsen EL, Jeon CO. Methane emission and dynamics of methanotrophic and methanogenic communities in a flooded rice field ecosystem. FEMS Microbiology Ecology. 2014 Apr 1;88(1):195–212.

36. Haroon MF, Hu S, Shi Y, Imelfort M, Keller J, Hugenholtz P, et al. Anaerobic oxidation of methane coupled to nitrate reduction in a novel archaeal lineage. Nature. 2013 Aug;500(7464):567–70.

37. Beal EJ, House CH, Orphan VJ. Manganese- and Iron-Dependent Marine Methane Oxidation. Science. 2009 July 10;325(5937):184–7.

38. Segarra KEA, Comerford C, Slaughter J, Joye SB. Impact of electron acceptor availability on the anaerobic oxidation of methane in coastal freshwater and brackish wetland sediments. Geochimica et Cosmochimica Acta. 2013 Aug 15;115:15–30.

39. Leu AO, Cai C, McIlroy SJ, Southam G, Orphan VJ, Yuan Z, et al. Anaerobic methane oxidation coupled to manganese reduction by members of the Methanoperedenaceae. ISME J. 2020 Apr;14(4):1030–41.

40. Al-Shayeb B, Schoelmerich MC, West-Roberts J, Valentin-Alvarado LE, Sachdeva R, Mullen S, et al. Borgs are giant genetic elements with potential to expand metabolic capacity. Nature. 2022 Oct;610(7933):731–6.

41. Schoelmerich MC, Ouboter HT, Sachdeva R, Penev PI, Amano Y, West-Roberts J, et al. A widespread group of large plasmids in methanotrophic Methanoperedens archaea. Nat Commun. 2022 Nov 18;13(1):7085.

42. Gao J, Yang J, Dong H, Tao S, Shi J, He B, et al. The origin of methyl group in methanogen-mediated mercury methylation: From the Wolfe cycle. Proc Natl Acad Sci USA. 2024 Oct 15;121(42):e2416761121.

43. Zhang X, Joyce GH, Leu AO, Zhao J, Rabiee H, Virdis B, et al. Multi-heme cytochrome-mediated extracellular electron transfer by the anaerobic methanotroph ‘Candidatus Methanoperedens nitroreducens.’ Nat Commun. 2023 Sept 30;14(1):6118.

44. Faust K, Lima-Mendez G, Lerat JS, Sathirapongsasuti JF, Knight R, Huttenhower C, et al. Cross-biome comparison of microbial association networks. Front Microbiol [Internet]. 2015 Oct 27 [cited 2024 Aug 21];6. Available from: https://www.frontiersin.org/journals/microbiology/articles/10.3389/fmicb.2015.01200/full

45. Faust K, Raes J. Microbial interactions: from networks to models. Nat Rev Microbiol. 2012 Aug;10(8):538–50.

# Supplementary Figures

**
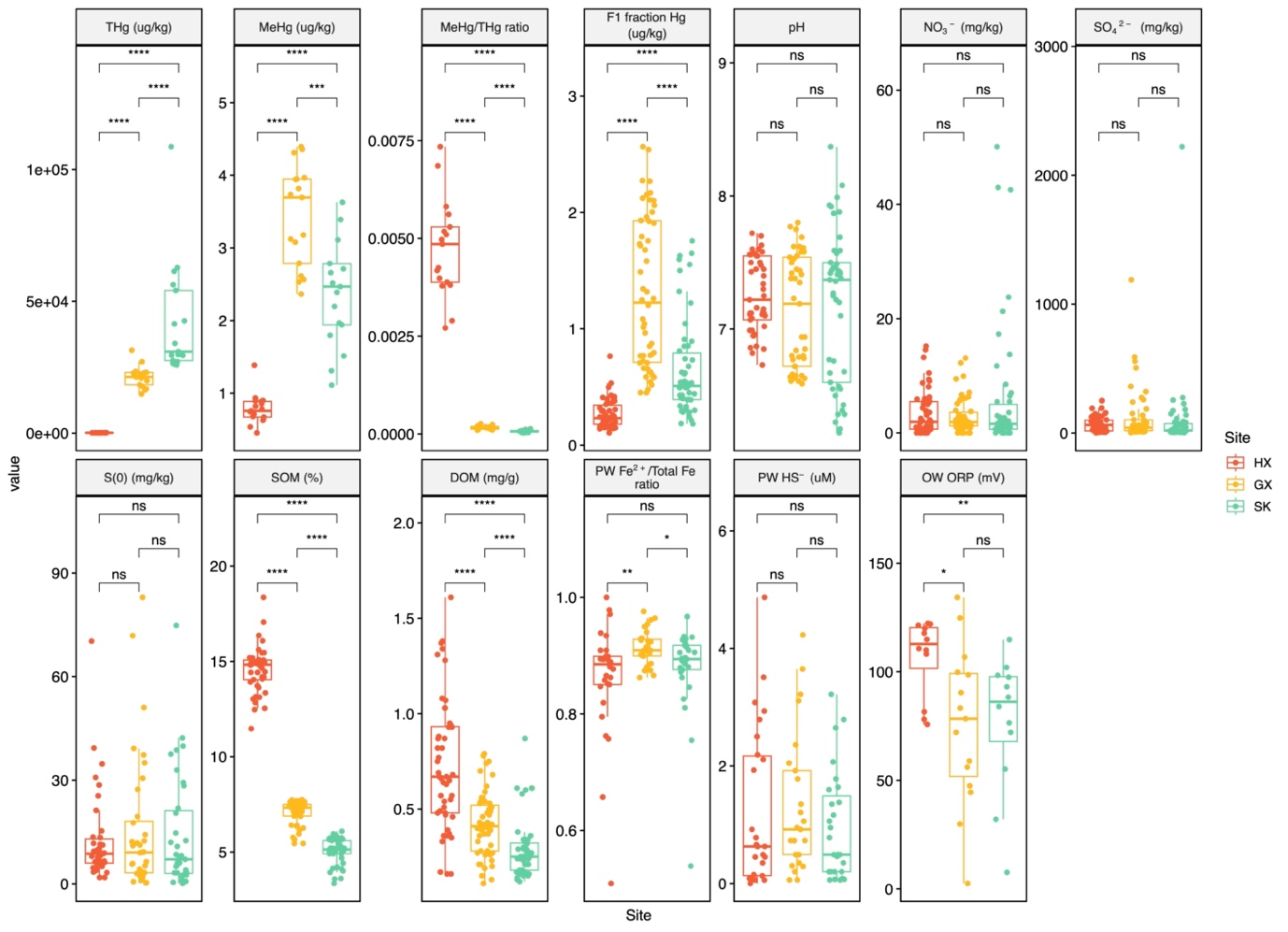
**

Figure S1. **Comparison of geochemical parameters among sites**. Boxplots illustrate the variation in geochemical parameters among the three sites (HX, GX, SK). Kruskal-Wallis post hoc pairwise comparisons are shown with significance levels indicated by asterisks: **** (𝑝 < 0.0001), *** (0.0001 ≤ 𝑝 < 0.001), ** (0.001 ≤ 𝑝 < 0.01), * (0.01 ≤ 𝑝 < 0.05), ns (𝑝 ≥ 0.05). Boxes represent the interquartile range (IQR) with medians indicated by horizontal lines. Whiskers extend to 1.5 times the IQR, and outliers are shown as points. Abbreviations: SOM (soil organic matter), DOM (dissolved organic matter), PW (porewater), OW (overlying water).


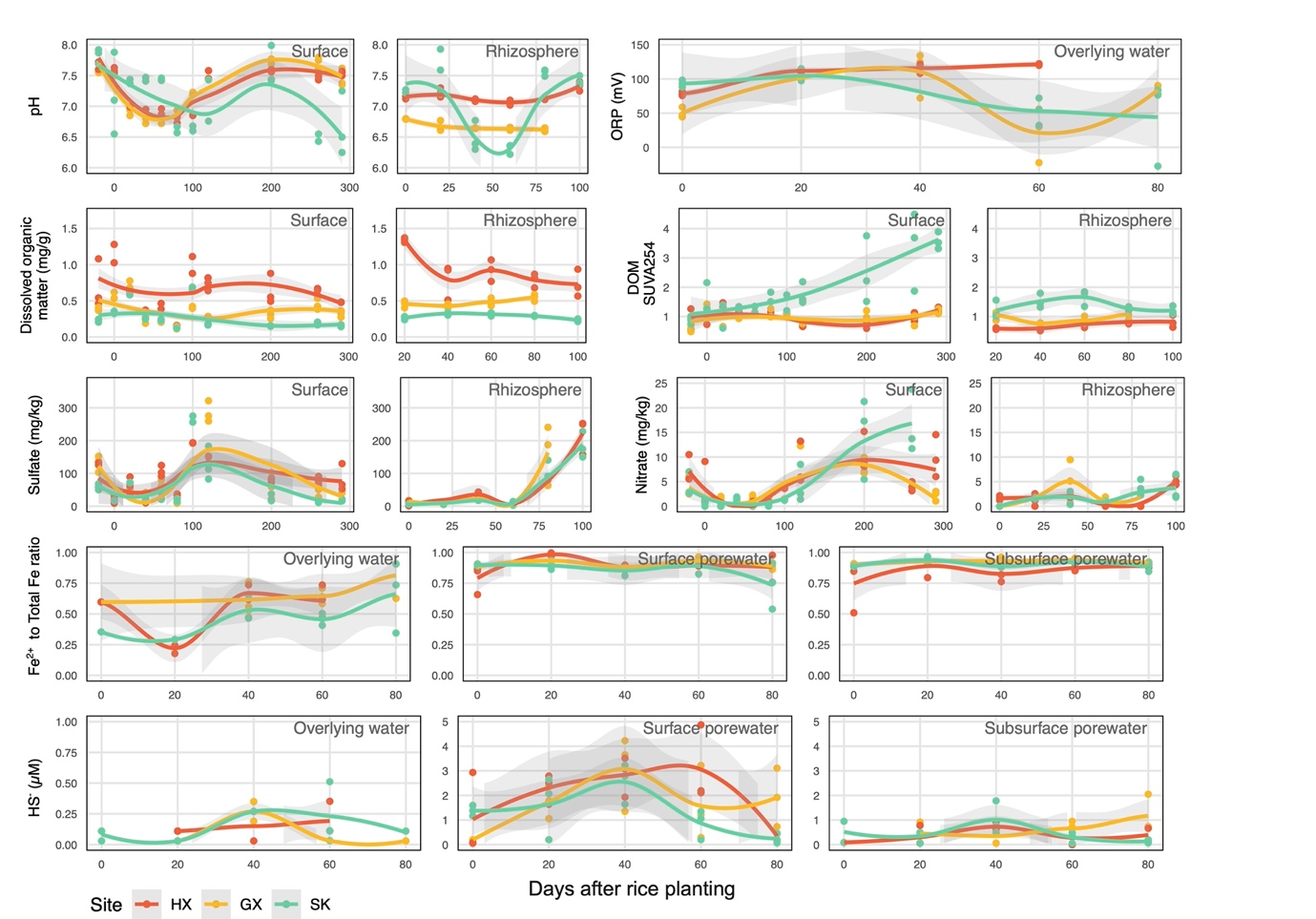


Figure S2. Temporal variation of geochemical parameters across three rice paddy sites. Geochemical measurements from surface soil, rhizosphere, overlying water, and porewater compartments include pH, oxidation-reduction potential (ORP), dissolved organic matter concentration and composition (DOM SUVA254), sulfate and nitrate concentrations, Fe²⁺/Total Fe ratio, and sulfide (HS⁻) concentrations. Data are presented with LOESS smoothing and 95% confidence intervals (shaded areas) where applicable; individual data points are shown. Note that ORP measurements represent overlying water conditions and do not reflect the more reduced conditions expected in soil microsites where anaerobic microbial processes occur.

Figure S3. **Correlations between F1 Hg, DOM and DOM-SUVA_254_ with MeHg in sediments across different sites and soil compartments.** Data are separated by site (HX, GX, SK) and soil compartment (Surface, Rhizosphere). Dashed lines indicate linear regression trends. Spearman's rank correlation coefficients (rho) and corresponding p-values are shown in the bottom right of each panel.

Figure S4. **Taxonomic distribution of key functional genes at the DNA and RNA levels in rice paddy samples at Day 60 after planting.** Stacked bar charts display the normalized abundance and taxonomic classification of five major functional genes: (A) hgcA (mercury methylation), (B) merB (mercury demethylation), (C) mcrA (methanogenesis), (D) pmoA/mmoX (methane oxidation), and (E) mbnT (methanobactin biosynthesis). For each gene, paired panels show metagenome data (DNA, left) and metatranscriptome data (RNA, right). Relative abundances are expressed as percentages of total reads (DNA) or mRNA reads (RNA), normalized by Hidden Markov Model (HMM) length. Bar colors represent taxonomic assignments at the Family, Phylum, or Genus level as indicated in the legend. Each horizontal gene pair allows direct comparison between gene presence (DNA) and expression (RNA) profiles.

Figure S5. **Temporal dynamics of Hg resistance gene (*merB*) abundance in rice paddies during the growing season.** The abundance of *merB* genes is shown in surface soils (left panel) and rhizosphere soils (right panel) across three sampling sites (HX, GX, SK). Data points represent individual measurements with LOESS smoothed trend lines illustrating temporal patterns. The *merB* gene abundance is expressed as percentage of total metagenomic reads normalized by hidden Markov model (HMM) length. Day 0 corresponds to the beginning of the sampling period. The results highlight site-specific variations in microbial Hg resistance potential in response to environmental conditions throughout the rice growing season.

Figure S6. **Relationship between methanogen and aerobic methanotroph abundances across different sites and soil types.** Scatter plot showing the standardized abundances (z-scores) of overall methanogens (x-axis) and overall aerobic methanotrophs (y-axis) from soil samples collected across three sites (HX, GX, SK) and two soil types (Surface, Rhizosphere). Each point represents an individual soil sample, with colors indicating sampling sites and shapes indicating soil types. The dashed black line represents the linear regression fit with 95% confidence interval (gray shading). Mixed-effects modeling revealed a significant relationship between methanogen and methanotroph abundances (β = 0.51, p = 0.00272, n = 50) after accounting for site and soil type.

Figure S7. **Taxonomic composition and abundance of the *mbnT* gene classified within specific methanotrophic genera previously implicated in MeHg demethylation in rice paddy soils throughout the growing season.** (A) The stacked bar chart illustrates the relative abundance of *mbnT* genes across different methanotrophic genera in both surface and rhizosphere soil samples collected from three distinct sites (HX, GX, SK). Data representation is expressed as the percentage of total metagenomic reads, normalized by hidden Markov model (HMM) length. Grey trend lines depict the temporal dynamics in total methanotroph abundance over time. The x-axis represents days post rice plantation. (B) Spearman correlation coefficients between MeHg concentration and *mbnT* gene abundance from selected methanotrophic genera across various sites and soil types are presented. Significance level indicates non-significant correlations (ns: not significant).


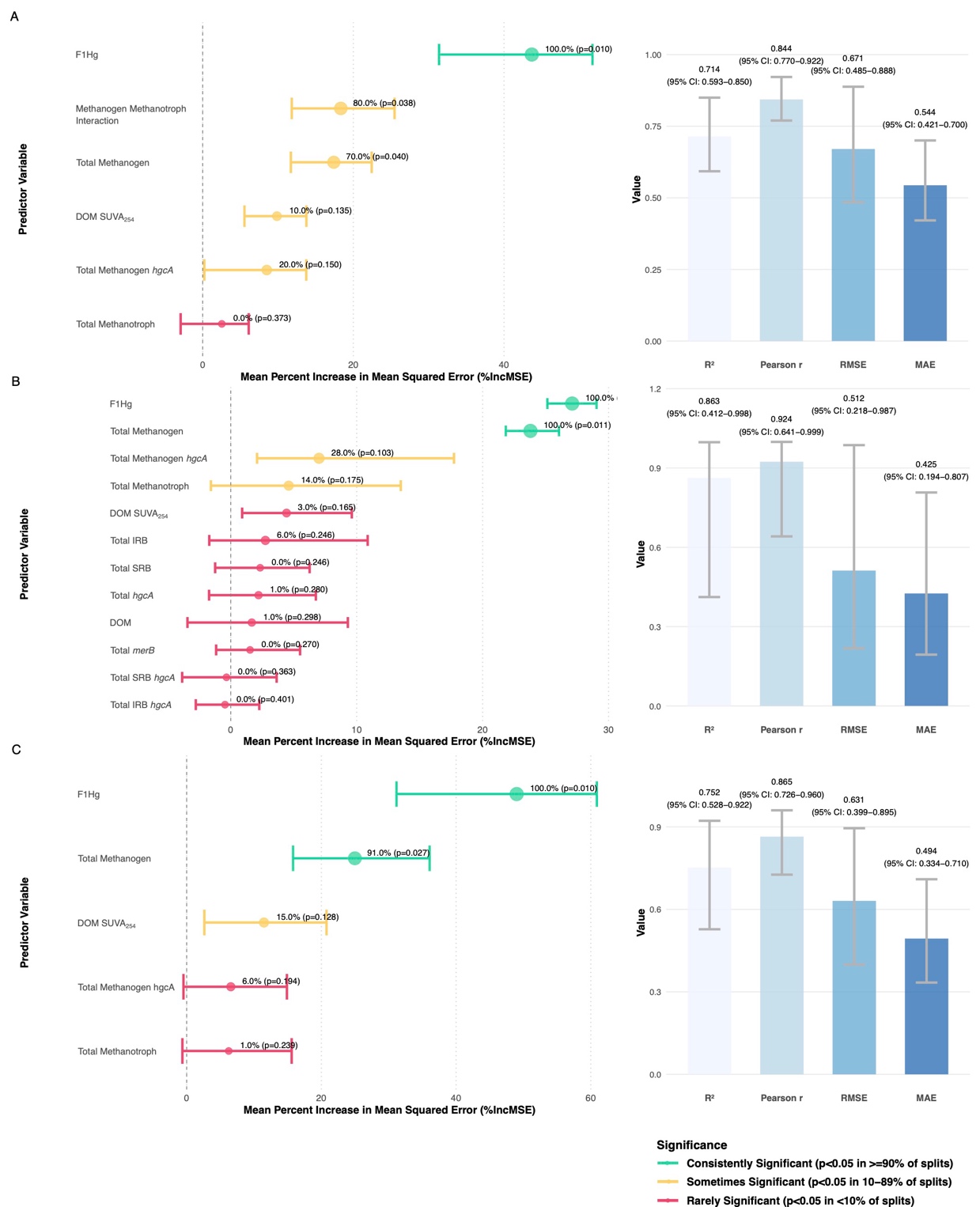


Figure S8. **Variable importance and model performance for predicting methylmercury (MeHg) concentrations using random forest analysis across three model configurations.** (A) Interaction model incorporating interaction terms between methanogens and methanotrophs, (B) flooded-period model (C) reduced model. For each model, variable importance plots (left panels) show the mean percent increase in mean squared error (%IncMSE) for each predictor variable when permuted, with 95% confidence intervals across 100 different data splits. Larger point size and green coloration indicate higher significance rates. Values display the percentage of data splits where the variable was significant (p<0.05) followed by the mean p-value. Model performance metrics (right panels) show mean values with 95% confidence intervals across all data splits. All models were validated through 100 permutation tests with random 70/30 train/test splits, with specific tree numbers and mtry parameters optimized for each model configuration.

Figure S9. Temporal dynamics of *Methanoperedenaceae* abundance in rice paddies during the growing season. The relative abundance of methyl-coenzyme M reductase (*mcrA*) genes from the *Methanoperedenaceae* family is shown in surface soils (left panel) and rhizosphere soils (right panel) across three sampling sites (HX, GX, SK). Data points represent individual measurements with LOESS smoothed trend lines illustrating temporal patterns. The *mcrA* gene abundance is expressed as percentage of total metagenomic reads normalized by hidden Markov model (HMM) length. Day 0 corresponds to the beginning of the sampling period.


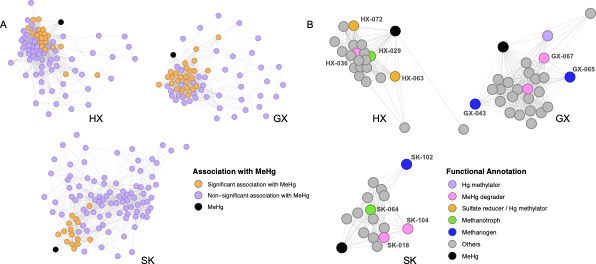


Figure S10**. Site-specific undirected network analysis of rice paddy microbial communities, based on Local Similarity (LS) scores derived from extended Local Similarity Analysis (eLSA), conducted across 50 metagenomic datasets. This analysis was used to predict associations between microorganisms and MeHg, as well as interactions with other community members, only significant interactions were retained from the eLSA analysis.** (A) Networks highlighting nodes with statistically significant associations with the MeHg node are presented for each site. (B) Partially filtered networks focusing only on nodes with significant associations to MeHg. Functional annotations are overlaid on these networks, with labels identifying nodes possibly involved in MeHg transformation.

Figure S11. **Site-specific microbial co-occurrence networks in the studied rice paddies.** This figure presents an undirected network analysis of rice paddy microbial communities based on Local Similarity (LS) scores derived from extended Local Similarity Analysis (eLSA) across 50 metagenomic datasets. The analysis predicts associations between microorganisms and MeHg, as well as broader microbial interactions, retaining only statistically significant connections. The networks depict phylum-level taxonomic classifications overlaid onto the network structure, with nodes representing microbial taxa and edges indicating significant associations. Key network properties, including average degree, density, and modularity, are provided for each site to characterize the structural organization of microbial interactions.
